# Biparental Care in a Southeast Asian Passerine, the Scarlet–Backed Flowerpecker (*Dicaeum cruentatum*)

**DOI:** 10.1002/ece3.71205

**Published:** 2025-04-02

**Authors:** Bridget Re, Dan H. Watson, Samantha N. Smith, Aubrey L. Alamshah, Surachit Waengsothorn, Max D. Jones

**Affiliations:** ^1^ Department of Fisheries and Wildlife Conservation Virginia Polytechnic Institute and State University Blacksburg USA; ^2^ Forestry and Environmental Conservation Department Clemson University Clemson USA; ^3^ Sakaerat Environmental Research Station, Thailand Institute of Science and Technological Nakhon Ratchasima Thailand

**Keywords:** breeding systems, flowerpecker family (*Dicaeidae*), life history, passerines, tropical ecology

## Abstract

We opportunistically observed a nest pair of scarlet‐backed flowerpeckers ( *Dicaeum cruentatum* ) for 5 days during the fledging period in late February 2020 within the Sakaerat Biosphere Reserve, Nakhon Ratchasima, Thailand. We observed both parents feeding the fledgling, with either parent returning within 3–20 min of leaving to forage. Over 5 days, both parents fed the fledgling green mistletoe fruits (*Dendrophthoe pentandra* [L.] Miq., family *Loranthaceae*). Between feeding periods, the fledgling remained mostly stationary on its perch. Our observations are similar to those reported for other species in the flowerpecker family (Dicaeidae), suggesting that biparental care is relatively common across the flowerpecker family, though there remains a paucity of direct observations and reporting. By making direct observations and subsequent discussions, we can better understand the ecology and natural history of *Dicaeidae* species, ultimately aiding in their conservation.

## Graphical abstract

Scarlet‐back flowerpeckers exhibit biparental care during the fledgling period. Both parents feed the fledgling green mistletoe fruits for a five‐day period within the Sakaerat Biosphere Reserve, Nakhon Ratchasima, Thailand.

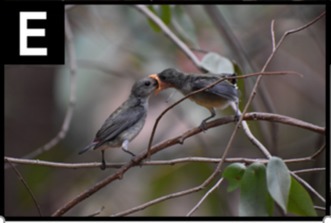

## 1 Introduction

The scarlet‐backed flowerpecker ( *Dicaeum cruentatum* ) is a passerine species in the flowerpecker family *Dicaeidae*. Scarlet‐backed flowerpeckers are thought to be common throughout the majority of their range in southern and Southeast Asia, but are considered rare in Bhutan and Nepal (BirdLife International 2023). They are found in subtropical and tropical moist lowland forests, wooded areas, and gardens generally below 1000 m, but have been documented at elevations as high as 1200 m in China and 2135 m in Nepal (Cheke and Mann 2020).

**FIGURE 1:**
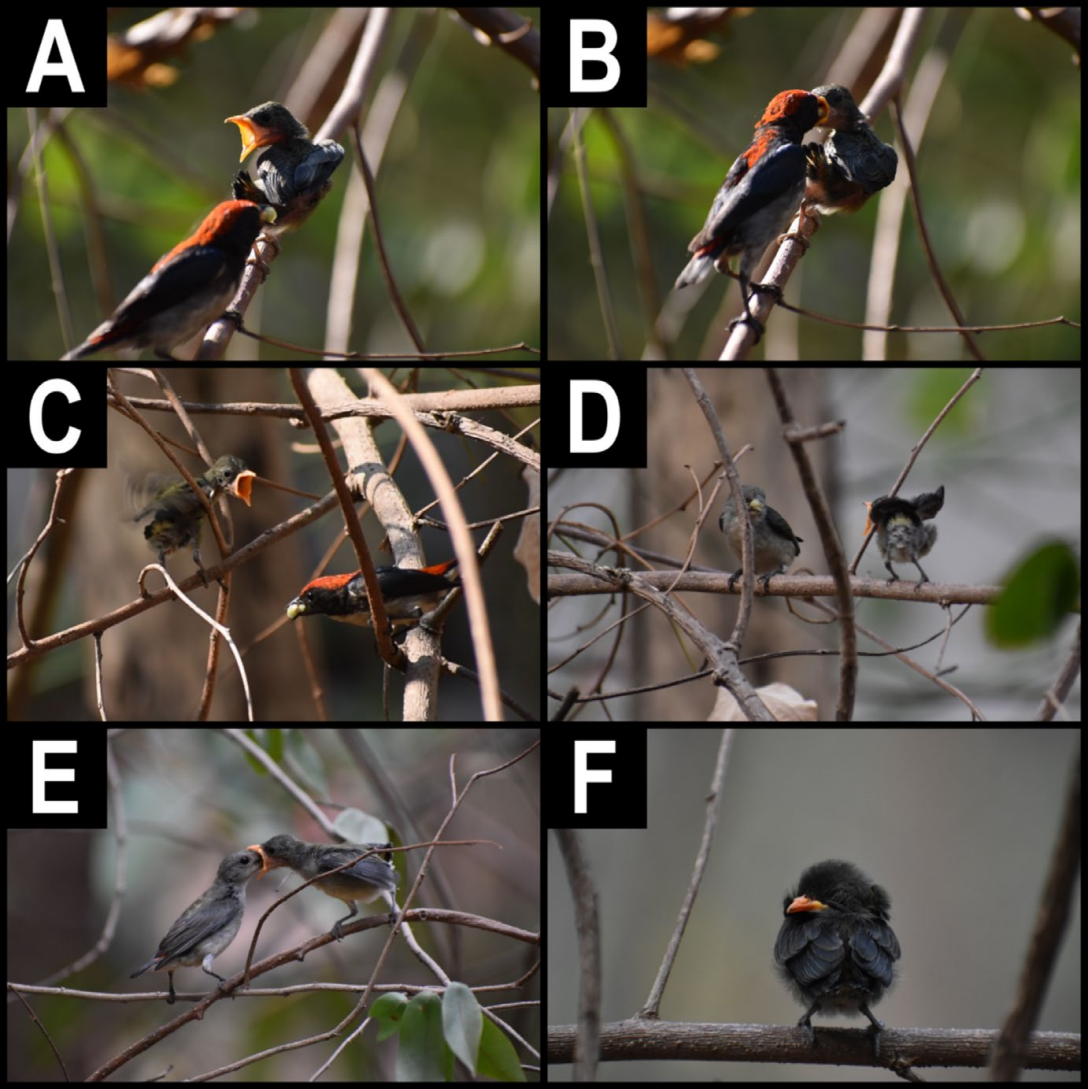
(A) A fledgling scarlet‐backed flowerpecker ( *Dicaeum cruentatum* ) about to be given a food item by a male parent—the adult male can be seen holding a green mistletoe fruit (*Dendrophthoe pentandra* [L.] Miq). (B) Male parent giving food item to fledgling; A and B depict the same feeding event. (C) Male parent about to give mistletoe fruit(s) to fledgling. (D) Female parent about to give mistletoe fruit to fledgling. (E) Female parent giving food item to fledgling. (F) Fledgling sleeping on perch. We estimated the fledgling to be approximately 5–7 cm tall.

Similar to most other species in the family *Dicaeidae*, scarlet‐backed flowerpeckers exhibit sexual dimorphism (Figure 1). Males have distinctive deep red colouration from the crown to the back and rump with glossy blue‐black wing and tail feathers (Figure 1A–C). Females are primarily drab gray‐brown with a red rump and black tail feathers (Figure 1D,E). Juveniles are similarly colored to females, but lack a red rump. Both sexes are known to incubate their clutch of 2–4 eggs as well as provision resources for nestlings and tend to fledglings (Cheke and Mann 2020). It is thought that most flowerpeckers form monogamous pairs during the breeding season, but this has not been extensively documented in the literature (Winkler et al. 2020). The diets of adult scarlet‐backed flowerpeckers are known to include *Muntingia calabura* and *Melastoma malabathricum* berries, figs (genus *Ficus*), mistletoes (family *Loranthaceae*), green seeds, nectar, and insects and spiders (order *Araneae*). Observations from scarlet‐backed flowerpecker nests in Malaysia documented the female stripping bark from a Makrut Lime tree ( *Citrus hystrix* ) and Pride of India (*Lagerstroemia* sp.) tree for nesting material and observed two nestlings being fed mistletoe (*Viscum* sp.) by a female (Amar‐Singh 2009; Chua et al. 2022). However, formal documentation of what adults feed their fledglings is lacking (Cheke et al. 2001; Cheke and Mann 2020).

In February 2020, an opportunity arose to formally document adult provisioning and closely observe parental care of a pair of scarlet‐backed flowerpeckers (*D. c. cruentatum*) within the Sakaerat Biosphere Reserve, Nakhon Ratchasima, Thailand.

### 1.1 Parental Care and Feeding of Fledgling

When we first discovered the fledgling in the afternoon on 25 February 2020, it was perched on a thin branch in full sunlight approximately 2 m above the ground, 500 m asl in mixed deciduous forest. During this initial sighting, both the male and female adult would leave and then return with food items for the fledgling (Table 1). We began recording using a Nikon D3200 (set on the “auto” function) from approximately 5 m away from the fledgling's perch. We recorded all subsequent video observations and took photographs from 5 m to minimize disturbance. During the beginning of the recording, we observed the fledgling continually chirping and preening itself. The fledgling stopped chirping and opened its mouth before the male flew into frame and fed it a green fruit, which we identified as belonging to the mistletoe species *Dendrophthoe pentandra* (L.) Miq. (*Loranthaceae*; Figure 1A–C). *D. pentandra* is a tropical woody shrub common throughout the Sakaerat Biosphere Reserve. We identified the plant species based on key morphological traits, including its small, fleshy berries that measure approximately 8–10 mm in length (Awang et al. Awang et al. 2023). Young fruits range from yellowish‐green to pink, while mature ones turn a deep red (Awang et al. Awang et al. 2023). To confirm our identification, we consulted a local flora expert who verified the species.

**TABLE 1:** Dates and times that we observed food items being fed to fledgling Scarlet‐backed Flowerpecker ( *Dicaeum cruentatum* ) at Sakaerat Biosphere Reserve, Nakhon Ratchasima, Thailand, February 2020.

Date	Time	Food item	Parent
25/2/2020	15:23	Mistletoe fruit	Male
25/2/2020	15:36	Mistletoe fruit	Male
25/2/2020	15:49	Mistletoe fruit	Male
25/2/2020	15:53	Mistletoe fruit	Male
25/2/2020	16:12	Mistletoe fruit	Female
26/2/2020	13:57	Unknown	Female
26/2/2020	14:11	Mistletoe fruit	Female
29/2/2020	19:02	Mistletoe fruit	Female
29/2/2020	19:07	Unknown	Female
29/2/2020	19:23	Unknown	Female

*Note:* Date format: Dd/m/yyyy. We were unable to record any observations on 27/2/2020 and 28/2/2020 because of being absent from the research station, and this does not likely reflect a lack of feeding by the parents.

After feeding the fledgling, the male departed immediately and the fledgling appeared to stay quiet for a few minutes before beginning to chirp regularly again. We observed this pattern multiple times: when an adult came into view, the fledgling would open its mouth; sometimes opening its wings and losing balance on the perch, before staying quiet after it had been fed. We observed both parents bringing fruit to the chick within 3–20 min of leaving to forage, with no discernible pattern, but seemingly in an opportunistic manner once a food item had been found (Figure 1A–E). Neither parent spent more than 4 s feeding the fledgling before departing to forage.

The following morning, the fledgling was observed in the same position on the branch sleeping with its head tucked backwards on top of its left wing (Figure 1F). We did not see either adult in the area and subsequent observations suggested it was a common occurrence for the fledgling to sleep alone within the vegetation where it was being fed. Biparental care during the post‐fledgling stage in other tropical species has been documented, with parents alternating between observing the fledgling from a high vantage point and foraging (Haddad et al. 2024). Although we did not observe this behavior directly, it is possible that the parents were monitoring the fledgling from a high vantage point outside our observation periods. During the second observation period, we noticed the fledgling moved to thinner branches with denser cover, approximately 1 m away from the initial feeding spot (Figure 1A–E). Similar to previous observations, we documented both parents feeding the fledgling every 4–13 min in an opportunistic manner. In one instance, we observed the male bring the chick two mistletoe berries (Figure 1C), though it was more common for a single food item to be presented. Between feeding periods, the chick chirped regularly and preened itself, occasionally rubbing its bill on small branches. Our observation periods were conducted opportunistically and lasted between 30 min to 2 h in the afternoon (Table 1). However, we frequently observed the chick sleeping in the morning. We continued to observe small‐scale movements and both parents feeding the fledgling mistletoe fruit until 29 February, after which we were unable to continue our observations.

## 2 Discussion

While we did not observe any arthropods being offered to the chick, they are known to be an important component of adult scarlet‐backed flowerpecker's diets and the *Dicaeidae* family in general (Cheke et al. 2001). Ballingall (1990) documented fruits being introduced into the diet after nestlings were 3 days old, but they did not take them until the fourth day. It is possible that arthropods are a component of nestling and fledgling scarlet‐backed flowerpecker's diets, but further observations are needed to determine when the parents introduce them into their diets. Mistletoe fruits are a known food item for scarlet‐backed flowerpeckers, and observations have been made at another site in Thailand of these birds manipulating mistletoe buds and probing flowers, potentially playing a role in the pollination of this plant (Cheke et al. 2001; Start 2013).

We were able to document important characteristics of parental care in scarlet‐backed flowerpeckers during our short observation period. Within the *Dicaeidae* family, formal documentation of parental care has been observed in a pair of Mistletoebirds (*Dicaeum hirundin*) in south‐east Queensland, Australia (Ballingall 1990). Similar to our observations, Ballingall noted both parents tending to fledglings, feeding them a mixture of fruit and arthropods. Ballingall observed the majority of the feeding conducted by the female, with the male starting feeding around mid‐morning and occasionally feeding until early afternoon. Both female and male Thick‐billed Flowerpeckers ( *Dicaeum agile* ) have been observed to partake in feeding nestlings and fledglings regularly (Katdare et al. Katdare et al. 2004), and both female and male Scarlet‐headed Flowerpeckers ( *Dicaeum trochileum* ) have been observed caring for nestlings (Taufiqurrahman 2011). However, at Blood‐breasted Flowerpecker ( *Dicaeum sanguinolentum* ) nests, Taufiqurrahman (2011) observed only the male tending to the nestlings. Thus, it appears that a variety of parental care strategies are exhibited by species in the *Dicaeidae* family, but it is not uncommon for biparental care to occur during the nestling and fledgling stages.

Bird species demonstrate various types of parental care during both pre‐ and post‐nestling stages. The most common form of parental care, observed in 81% of species, is biparental care, where females and males cooperate to raise offspring (Cockburn 2006). Due to the commonality of biparental care, it is less likely to be studied and reported on (Cockburn 2006), especially for tropical species where more conspicuous forms of breeding attract the attention of researchers (i.e., lekking systems, cooperative breeding; Macedo 2008). There is both a geographic bias of nesting studies (Ibáñez‐Álamo et al. 2015) as well as a scarcity of data from tropical regions despite the high biodiversity within these systems. Our findings contribute insights into the parental care strategies employed within the *Dicaeidae* family. By examining variations in life history strategies across species, we can deepen our understanding of their ecology and inform future conservation efforts.

## Author Contributions

**Bridget Re:** investigation (equal), writing – original draft (lead), writing – review and editing (equal). **Dan H. Watson:** investigation (equal), writing – review and editing (equal). **Samantha N. Smith:** conceptualization (equal), investigation (equal), writing – review and editing (equal). **Aubrey L. Alamshah:** conceptualization (equal), investigation (equal), writing – review and editing (equal). **Surachit Waengsothorn:** validation (equal), writing – review and editing (equal). **Max D. Jones:** conceptualization (equal), data curation (lead), investigation (equal), supervision (lead), visualization (lead), writing – review and editing (equal).

## Conflicts of Interest

The authors declare no conflicts of interest.

## Data Availability

All data are included in Figure 1 and Table 1.
